# Evidence-Based Priority Setting for Health Care and Research: Tools to Support Policy in Maternal, Neonatal, and Child Health in Africa

**DOI:** 10.1371/journal.pmed.1000308

**Published:** 2010-07-13

**Authors:** Igor Rudan, Lydia Kapiriri, Mark Tomlinson, Manuela Balliet, Barney Cohen, Mickey Chopra

**Affiliations:** 1 Global Health Academy and Centre for Population Health Sciences, The University of Edinburgh Medical School, Edinburgh, Scotland, United Kingdom; 2 Croatian Centre for Global Health, Faculty of Medicine, University of Split, Soltanska, Split, Croatia; 3 Department of Health, Aging and Society, McMaster University, Hamilton, Ontario, Canada; 4 Department of Psychology, Stellenbosch University, Stellenbosch, South Africa; 5 Committee on Population, United States National Academy of Sciences, Washington, D.C., United States of America; 6 UNICEF, New York, New York, United States of America

## Abstract

As part of a series on maternal, neonatal, and child health in sub-Saharan Africa, Igor Rudan and colleagues discuss various priority-setting tools for health care and research that can help develop evidence-based policy.

This paper is part of a *PLoS Medicine* series on maternal, neonatal, and child health in Africa

## Priority Setting—Implicit or Explicit?

Priority setting is required in every health care system. It guides investments in health care and health research, and respects resource constraints. It happens continuously, with or without appropriate tools or processes. Although priority-setting decisions have been described as difficult, value laden, and political, only a few research groups are focused on advancing the theory of priority setting and the development and validation of priority setting tools [1]–[4]. These groups advocate the use of their tools, but their work is often not widely recognized, especially among the policy makers in developing countries, where these tools would be most helpful [2].

Our primary objective in this essay is to present the available tools for priority setting that could be used by policy makers in low-resource settings. We also provide an assessment of the applicability and strengths of different tools in the context of maternal and child health in sub-Saharan Africa.

The analyses of investments in neglected diseases showed that they lack transparent priority-setting processes [2]. This persisting situation results in remarkable levels of inequity between investments in different health priorities [1]–[6]. Therefore, our secondary objective is to advocate for the use of the tools that could lead to more rational priority setting in sub-Saharan Africa. An optimal tool should be able to draw on the best local evidence and guide policy makers and governments to identify, prioritize, and implement evidence-based health interventions for scale-up and delivery.

## Priority Setting in Low-Resource Settings—Mixed Evidence

Although there is currently insufficient evidence that the use of priority-setting tools improves health outcomes and reverses existing inequities, we have ample evidence that the lack of a rational and transparent process generates inequity and stagnation in mortality levels [5],[6]. Recently, Youngkong et al. conducted a systematic review of empirical studies on health care priority setting in low-income countries (Table 1) [7]. The review found that policy makers in developing countries rarely consider using the available priority-setting tools, but also that the available tools lack credibility for priority setting in low-resource settings [7],[8]. This is mainly because it is not easy to validate the tools or to link their output with concrete follow-up actions and policy development [9]. Indeed, it is difficult to prove beyond all doubt that investments in health care or health research are valuable to society when compared to alternative investments such as infrastructure or the economy.

**Table 1:** Priority setting exercises for health care or health research in low resource settings.

Setting	Participants	Topic	Criteria	Process	Outcome
**Health care/health interventions – all low-resource countries (Refs. ** [**13**],[21],[**22**] **)**					
Low-resource globally	TE	All major diseases	DCPP project consensus	Systematic reviews	Cost-effectiveness analysis
Low-resource globally	TE, PM, OS	Primary health care	Yes, modified CHNRI	CHNRI	Specific list with scores and ranks
Low-resource globally	TE, PM, OS	Stillbirth prevention	Yes, modified CHNRI	CHNRI	Specific list with scores and ranks
**Health care/health interventions - national or sub-national level (Refs. ** [**7]**,[34],[35],[37****] **)**					
Thailand	PM, HM, HW, TE	Several diseases	Yes, through literature review	Semi-structured Interview	Table with choice frequency
Chile	None	Health system	Yes, through literature review	Secondary data analysis	List with ranks for 56 choices
South Africa	PM, NGO, TE	HIV/AIDS	Yes, through literature review	Group discussion and interview	List with ranks by THREE chosen criteria
Tanzania	PM	Health system	Yes, through group discussions	Group discussion and question.	Ranking of criteria by importance
Tanzania	PM, HP, GP, PA	Health system	Not transparent	Group discussion	Description of different views
Tanzania	PM, HP, GP, PA	Several diseases	Yes, through literature review	Deliberative process	List with ranks for NINE interventions
Argentina	PM (at all levels)	Health system	Yes, focus group and interviews	Focus group and interviews	List of criteria
Nepal	PM, HP	Several diseases	Yes, literature review and group discussions	Individual rating	List with ranks for 33 interventions
Pakistan	PM	HIV/AIDS	Yes, in-depth interview	Interview	Description of policy maker's views
Burkina Faso, Ghana, Indonesia	PM, HM, HW	Safe motherhood program	Yes, self-administered questionnaire	Deliberative process and quest.	Identifying three most important priorities
Uganda	PM, HW	Health system	Yes, interviews	Semistructured interview	Description of criteria used
Uganda	PM, HW	Health system	Yes, one-on-one interviews	Interview and document analysis	Description of criteria used
Ghana	PM, HW	Several diseases	Yes, group discussion	Individual rating	List with ranks for interventions
Uganda	PM, HW, GP	Health system	Yes, literature and self-administered questionnaire	Questionnaire with rating scale	List of criteria and their weights
Uganda	PM, HW, GP	Health system	Yes, group discussions and interviews	“Brainstorming” and questionnaire	List with ranks for criteria and choices
Ghana	PM, HM, TE, NGO	Reproductive health	Yes, literature review and interview	Interview and secondary data	Demonstration of impact on priorities
Bosnia and Herzegovina	None	Health system	Not transparent	Secondary data analysis	Description of criteria used
South Africa	None	Health system	Yes, literature review	Secondary data analysis	List with ranks for interventions
India	PM, TE	Neonatal mortality	Yes, literature review and model	Lives Saved Tool (LiST)	Effectiveness and impact on mortality
Ghana and Mali	PM, TE	Child mortality	Yes, literature review and model	Lives Saved Tool (LiST)	Effectiveness and impact on mortality
Burkina Faso, Ghana, Malawi	PM, TE	Child mortality	Yes, literature review and model	Lives Saved Tool (LiST)	Effectiveness and impact on mortality
**Health research – all low-resource countries (Refs. ** [**23]**–[29****] **)**					
Low-resource globally	TE, PM, HP, OS	Mental health	Yes, standard CHNRI	CHNRI	Specific list with scores and ranks
Low-resource globally	TE, HP	Maternal and child survival	None; collective opinion	Delphi	Specific list of priorities with ranks
Low-resource globally	TE, PM, HP, OS	Neonatal infections	Yes, standard CHNRI	CHNRI	Specific list with scores and ranks
Low-resource globally	TE, PM, HP, OS	Childhood diarrhea	Yes, standard CHNRI	CHNRI	Specific list with scores and ranks
Low-resource globally	TE, PM, HP, OS	Birth asphyxia	Yes, standard CHNRI	CHNRI	Specific list with scores and ranks
Low-resource globally	TE, PM, HP, OS	Childhood pneumonia	Yes, standard CHNRI	CHNRI	Specific list with scores and ranks
Low-resource globally	TE, PM, HP, OS	Zinc supplementation	Yes, standard CHNRI	CHNRI	Specific list with scores and ranks
Low-resource globally	TE, PM, HP, OS	Research into disabilities	Yes, modified CHNRI	CHNRI	Specific list with scores and ranks
**Health research – national or sub-national level (Refs. ** [**30**],[**31**] **)**					
Malaysia	TE, PM, OS	Health research	Yes, transparent list	CAM	General recommendations
Cameroon	Government officials	Health research	Not transparent	ENHR and COHRED	General objectives
Peru	TE	Health research	Not transparent	COHRED	General recommendations
South Africa	Government officials	Health research	Yes, transparent	ENHR	General recommendations
South Africa	TE, OS	Child health research	Yes, standard CHNRI	CHNRI	Specific list with scores and ranks
Brazil	PM, TE, HP, multiple OS	Health research	Yes, transparent	COHRED	General recommendations
Philippines	PM, TE, HP, OS	Health research	Not fully transparent	COHRED	General recommendations
Pakistan	PM, TE, HP, NGO, PS	Health research	Yes, transparent	CAM	General recommendations
Argentina	PM, TE, HP	Health research	Yes, transparent	CAM	General recommendations

**Abbreviations:** GP, general population; HM, health managers; HP, health professionals; HW, health workers; NGO, non-governmental organization; OS, other stakeholders; PA, patients; PM, policy-makers; PS, private sector; TE, technical experts.

However, there are many examples of countries that have reduced their maternal and child disease burden substantially from very high starting levels, and of others that keep failing to achieve progress [10]. We also have strong evidence on the key determinants of those successes, which has been incorporated into various priority-setting tools [1],[4]–[9]. The few studies that have evaluated processes in low-resource settings not using priority-setting tools found that most of them fell short on all four conditions of the “accountability for reasonableness” framework that assessed their basic legitimacy and fairness [11],[12].

Moreover, there is evidence on the interventions and health research needed to improve maternal and child survival in low-resource settings. The key challenge is how to motivate and educate policy makers in sub-Saharan Africa to use the available priority-setting tools to direct the limited available resources into the most effective interventions and health research. We believe that addressing this challenge is critical, because it has been repeatedly shown that the scarcity of resources for health in sub-Saharan Africa is only part of the larger problem; the other part is that the scarce available resources are not being used efficiently by any standard, leading to tragic consequences for the population [2],[4],[6].

## Emerging Tools for Evidence-Based Priority Setting to Guide Health Care Policy

Several tools and processes are beginning to emerge as useful for priority setting in low-resource settings. In Table 1 we classify different methodologies by the context (national/global level) and scope (health care/health research prioritization). We also provide some essential information on the use of each method: (i) the setting; (ii) participants included in the process; (iii) the specific topic addressed; (iv) the criteria that were used for prioritization; (v) the process that was used; and (vi) the nature of the outcome. An in-depth comparative analysis of all these tools is beyond the scope of this essay, but in Table 1 we provide references to the key papers from which further information about those methods can be obtained ([13]–[37]; Lawn et al., manuscript in preparation).

Table 1 shows that the “burden of disease/cost effectiveness analysis,” promoted by the Disease Control Priorities Project (DCPP) [13], is an essential component of several tools that have been used for health care (interventions) prioritization: for example, the Marginal Budgeting for Bottlenecks (MBB) tool developed by UNICEF and The World Bank [14]; WHO-CHOICE (Choosing Interventions that are Cost-Effective) developed by the World Health Organization [14],[15]; and Lives Saved Tool (LiST) developed by Johns Hopkins University scientists and the Futures Institute [16]. The DCPP approach for developing countries uses information on the burden of major diseases to assist decisions about the potential of affordable and effective interventions. The DCPP analysis identifies the “best buys,” i.e., the most cost-effective interventions in terms of DALYs saved per unit cost, that should compose a country's essential health care package (EHCP) [17]. The EHCP should then influence program design and resource reallocation to help governments achieve the goal of reducing morbidity and mortality.

However, the DCPP authors note that factors other than cost-effectiveness influence priority setting in the real world, so the available evidence has to be considered in the context of local realities [13],[17]. Both MBB and WHO-CHOICE provide appropriate contextualization tools. However, the LiST software goes much further than any other tool in several dimensions. LiST contains an expansive evidence base of context-specific intervention effectiveness, generated by researchers from the WHO/UNICEF's Child Health Epidemiology Reference Group (CHERG) [33]. It is a user-friendly decision-making computer software available in the public domain. It enables estimation of intervention impact on child mortality at national, regional, and global levels [16]. Further important advantages of LiST include its validation in both African and South Asian contexts [34],[35], an ability to perform very specific comparisons between alternative investment strategies over a specified time frame in terms of child survival outcomes [33]–[35], its application of an equity lens [36], and easy translation of outcomes into program planning with convincing country-level examples [37].

## Prioritizing Gaps in Health Research

Policy makers in low-resource settings also need to set priorities for health research. Table 1 shows that the CHNRI methodology has recently been used by several different groups to set health research priorities at the highest international level ([23]–[29], Lawn et al., manuscript in preparation). However, there are several other tools for setting research priorities at the national level, which were reviewed and evaluated by Tomlinson et al. [30]. Whereas CHNRI method had its first national-level implementation in South Africa only recently [31], other tools and processes have been dominant at the national level. The Council on Health Research for Development's approach (COHRED) has been implemented in Brazil, Cameroon, Peru, and Philippines; the Essential National Health Research (ENHR) approach in Cameroon and South Africa; and the Combined Approach Matrix (CAM) in Malaysia, Pakistan, and Argentina [30].

COHRED, ENHR, and CAM all were developed by committees set up by international agencies. All these methods are very specific about context, and they are excellent for organizing all the available information. However, they do little to provide an algorithm, based on a transparent set of criteria, that can distinguish among many competing research investment options [4],[29]. This does not, however, diminish their utility in most situations where the development of an evidence base is required. That phase can then be followed by Delphi-type consultation processes among a designated set of experts. For example, CAM does exceptionally well in addressing the two dimensions of the context that it finds the most important: the “public health” dimension and the “institutional” dimension. Having only two dimensions limits CAM's flexibility, though, and it is difficult to see how additional dimensions—e.g., uncertainty over the outcome (inherent to all health research); accounting for investment styles; accounting for the risk exposure and benefit potential of each research option; or the likelihood of obtaining funding support from donors—could be added [33]. The same limitation is also true for COHRED and ENHR.

An emerging tool that is rapidly gaining popularity in the area of health research prioritization is the CHNRI methodology. It was developed over four years (2005–2008) with support from The World Bank for a transdisciplinary exercise of 15 experts. The experts assessed principles and practice of priority setting [4], reviewed universal challenges [18], developed a novel and robust conceptual framework [18], and provided guidance for stakeholder involvement [19] and for implementation of the method [20]. Currently, they are in the process of developing user-friendly software that would enable simple, cheap, and effective conducting of CHNRI exercise via the internet.

The CHNRI methodology insists on transparency about the context in which priority setting takes place and the criteria used. It was initially developed for health research, but it has recently also been successfully used for health care and health interventions (Table 1) [21],[22]. Like the DCPP approach, it uses both cost-effectiveness and potential impact on disease burden as criteria. However, within a set of “standard” criteria, CHNRI also uses criteria relevant to the context—answerability, deliverability, affordability, sustainability, local capacity, likelihood of support, feasibility, equity, and others. The process is usually designed by policy makers or donors, conducted by technical experts in a transparent way (e.g., each vote counts equally), with a mechanism of stakeholder involvement. Stakeholders can assign different weights to the criteria used in the CHNRI exercise. The outcome is a comprehensive list with competing priorities ranked according to the combined scores they received in the process [18]–[20]. Such a list is helpful to policy makers because it provides an overview of strengths and weaknesses of competing investment options against many criteria, based on the collective input of technical experts. The list can also be adjusted by taking the values of many stakeholders into account.

## Conclusions

The key challenges that need to be overcome in sub-Saharan Africa to improve the processes of prioritization in health care and health research include the following: increased acceptability and popularity with local policy makers, appreciation of the local context, clarity about the criteria used, transparency in the input from the stakeholders, and more specific guidance on translation into policy. Many papers that analyze the strategies for improving maternal and child survival conclude with highlighting the challenges such as integration, requirements for selection of community health workers, operational research into systems, among others. These are all admirable and important future areas of research. However, they are not exactly new, ground-breaking, or very specific, and the qualitative nature of the process frequently does not provide sufficient guidance to policy makers on the specific next steps. Tools such as LiST (for health care/interventions) and CHNRI (for health research) involve local experts and incorporate issues of local context into priority determination in a transparent, user-friendly, replicable, quantifiable and specific, algorithm-like manner. Both of these tools were primarily developed to address child health problems and should be considered by policy makers in the area of maternal and child health in sub-Saharan Africa.

The use of scientific evidence and principles in setting health priorities has an enormous potential to lead to more rational decision making, especially in low-resource settings where decision making has long lacked formal tools, processes, or an evidence base. We believe one cannot overstate the value of building and supporting the capacity of local experts and policy makers in sub-Saharan Africa to initiate and assist their own national government's policy formation process in maternal and child health, and of government's being able to generate rigorous credible “home grown” advice [4],[27],[32]. Regardless of the limitations of the available tools, we strongly recommend their use in development of sound maternal and child health policies in sub-Saharan Africa over the alternative of not using any method. The use of such tools would promote attention to objective evidence in public policy debates, often leading to decisions that are made are more clearly and in the public interest [27],[32].

## Abbreviations

CAM: Combined Approach Matrix
CHNRI: Child Health and Nutrition Research Initiative
CHOICE: Choosing Interventions that are Cost-Effective
COHRED: Council on Health Research for Development
DALY: disability-adjusted life year
DCPP: Disease Control Priorities Project
EHCP: Essential Health Care Package
ENHR: Essential National Health Research
LiST: Lives Saved Tool
MBB: Marginal Budgeting for Bottlenecks
WHO: World Health organization
